# Pharmacists’ role in recognising medication-related oral health risks in aged care: a qualitative study of pharmacists’ and dentists’ perspectives

**DOI:** 10.1007/s11096-026-02225-w

**Published:** 2026-09-16

**Authors:** Joon Soo Park, Amy T. Page, Estie Kruger, Woosung Sohn, Bradley Christian, Arash Rudman

**Affiliations:** 1 School of Health and Clinical Sciences, The University of Western Australia, Crawley, WA Australia; 2 School of Dentistry, Faculty of Medicine and Health, The University of Sydney, Camperdown, NSW Australia; 3 Australian Centre for Integration of Oral Health, School of Nursing and Midwifery, Western Sydney University, Sydney, Australia; 4 Westmead Applied Research Centre, Faculty of Medicine and Health, The University of Sydney, Camperdown, NSW Australia

**Keywords:** Aged care, Interdisciplinary care, Medication management, Oral health, Polypharmacy, Qualitative research

## Abstract

**Introduction:**

Medication-related oral health complications, including salivary gland hypofunction, xerostomia, dental caries, candidiasis, mucosal injury, and swallowing difficulties, are common among older adults exposed to polypharmacy across aged-care and broader clinical practice contexts. Pharmacists are increasingly involved in medication management in residential aged care and may be well positioned to support the earlier recognition of medication-related oral health risks. Despite this, previous literature suggests that oral health is often insufficiently integrated into medication management, interdisciplinary care pathways, and pharmacist education frameworks.

**Aim:**

This study aimed to explore pharmacists’ and dentists’ perspectives on pharmacists’ role in recognising medication-related oral health risks in aged-care practice, including pharmacists’ educational needs, interdisciplinary care considerations, and preferred implementation strategies for practical oral health resources.

**Method:**

A qualitative descriptive study was conducted in Australia using semi-structured interviews with pharmacists and dentists who had experience caring for older adults, managing polypharmacy, or working in aged-care-related practice. Participants were recruited using purposive sampling to capture a range of clinical perspectives across pharmacy and dental settings. Interviews were audio-recorded, transcribed verbatim, and analysed using reflexive thematic analysis.

**Results:**

Three major themes were identified from thirteen participants (six dentists and seven pharmacists): limited formal preparation for medication-related oral health risks; reactive recognition of oral complications within fragmented care systems; and the need for practical, workflow-integrated resources to support pharmacists’ involvement in oral health care. Participants described limited oral health education, delayed recognition of oral complications, unclear referral pathways, and poor interdisciplinary communication. Xerostomia was the most commonly recognised symptom, although participants demonstrated less consistent recognition of objective salivary gland hypofunction and its consequences, including dental caries, candidiasis, swallowing difficulties, and nutritional decline. Concise educational resources, decision-support tools, and electronic prompts were strongly preferred to support earlier recognition and referral within medication management workflows.

**Conclusion:**

Pharmacists and dentists described educational, interdisciplinary, and systems-level challenges affecting pharmacists’ involvement in recognising and managing medication-related oral health risks in aged-care practice. Findings suggest that practical educational resources, clearer referral pathways, and stronger interdisciplinary collaboration may support pharmacists’ involvement in recognising medication-related oral health risks among older adults with polypharmacy.

**Supplementary Information:**

The online version contains supplementary material available at https://doi.org/10.1007/s11096-026-02225-w.

## Impact statements

- Participants described medication-related oral health complications as insufficiently recognised within their respective clinical practice contexts involving older adults, particularly among older adults exposed to polypharmacy. Limited formal education, fragmented referral pathways, and poor interdisciplinary integration were described as barriers to pharmacists’ involvement in recognising and responding to oral complications.
- Participants preferred concise, workflow-integrated educational resources, including checklists, decision-support tools, and electronic prompts. Participants also highlighted the importance of clearer referral pathways and stronger interdisciplinary collaboration to support pharmacists' role in recognising and communicating medication-related oral health risks within medication management.
- Findings suggest that practical oral health resources and greater integration of oral health education within medication review and Aged Care On-site Pharmacist workflows may support pharmacists working with older adults.

## Introduction

Medication-related oral health complications are increasingly recognised as an important yet under-addressed issue among older adults, particularly those experiencing multimorbidity and polypharmacy. Older people commonly experience multimorbidity and polypharmacy, with many exposed to medicines associated with xerostomia, salivary gland hypofunction, candidiasis, mucosal irritation or ulceration, dysphagia, dental caries, and periodontal complications [1, 2]. These complications can substantially impair oral function, nutrition, communication, and quality of life, while also contributing to broader systemic consequences such as frailty, infection risk, and reduced overall well-being [3–5]. Despite growing awareness of oral-systemic interactions, that is, the bidirectional relationships through which systemic diseases and medicines affect oral health, and oral conditions may influence general health, medication-related oral health risks remain insufficiently integrated into routine medication management [6, 7].

Pharmacists may be well-positioned to contribute to the recognition and management of medication-related oral complications due to their central role in medication review, deprescribing, and quality use of medicines initiatives. Recent Australian reforms, including the Aged Care On-site Pharmacist programme, have expanded pharmacists’ responsibilities within residential aged-care homes and may create opportunities to integrate oral health considerations into routine medication management [8–10]. However, existing pharmacist education and training frameworks provide limited guidance on recognising medication-related oral complications, communicating oral health concerns, and referring patients to dental professionals [11]. Pharmacist–dentist communication is not routinely embedded within medication review or dispensing pathways, and pharmacists may have limited direct contact with dental professionals when medication-related oral concerns are identified. Communication may instead occur indirectly through patients, general practitioners, nurses, carers, or aged-care staff, creating uncertainty regarding referral responsibilities and follow-up [12, 13].

Implementation research suggests that effective educational interventions in aged care must align with real-world clinical workflows and organisational constraints [14, 15]. Pharmacists working in aged-care settings frequently encounter time pressures, workforce and organisational constraints, and complex clinical presentations [8–10, 14, 16], highlighting the need for concise, practical, and workflow-integrated educational resources. While previous research has examined oral health in aged care and pharmacist roles in medication review, less is known about how pharmacists and dentists understand medication-related oral health risks, what educational support pharmacists require, and how such resources could be implemented within aged-care workflows [16, 17]. Addressing this gap is important as pharmacists take on expanded roles in residential aged-care homes through medication review and Aged Care On-site Pharmacist programme-related services [11].

### Aim

This study aimed to explore pharmacists’ and dentists’ perspectives on pharmacists’ potential role in recognising and responding to medication-related oral health risks among older adults. The objectives were to: identify perceived gaps in pharmacists’ preparation for recognising medication-related oral health risks; compare pharmacists’ and dentists’ perspectives on interdisciplinary communication, referral pathways, and professional scope; and examine preferred formats and implementation considerations for practical oral health resources within medication management workflows.

## Method

### Study design

This qualitative descriptive study used semi-structured interviews to explore pharmacists’ and dentists’ perspectives on pharmacists’ role in recognising and managing medication-related oral health risks among older adults in aged-care practice. The study was reported in accordance with the Consolidated Criteria for Reporting Qualitative Research (COREQ) checklist [18].

### Philosophical positioning

This study was informed by a constructivist/interpretivist paradigm [19], recognising that participants’ perspectives are shaped by their professional and personal experiences [20].

### Rigour and trustworthiness

JP, a male Australian board-registered dentist and pharmacist working as a senior research fellow, conducted all one-on-one semi-structured interviews. His clinical and research experience in oral health, pharmacotherapy and aged-care practice informed the interview guide and interpretation of findings. Reflexivity was supported through post-interview notes, awareness of potential disciplinary biases, and the use of open-ended questions and neutral prompts. Emerging codes, themes and interpretations were discussed regularly with EK and AR to challenge assumptions and reduce single-researcher interpretive bias.

### Participants

Participants were eligible if they were registered pharmacists or dentists currently practising in Australia and involved in aged-care practice, medication management, oral healthcare, or the care of older adults experiencing polypharmacy. Pharmacists were included because of their role in medication review, medicine-related risk assessment, and communication with prescribers. Dentists were included to provide complementary clinical perspectives on the oral manifestations and consequences of medication-related complications, as well as referral and interdisciplinary communication processes. The study focused specifically on the pharmacist–dentist interface; other aged-care professionals were outside the study scope. Participants were recruited through targeted advertisements and professional networking posts on Facebook. Recruitment was purposive in that advertisements specifically sought pharmacists and dentists with experience in older-adult care, polypharmacy, medication review, aged care, or medication-related oral health. However, participation was voluntary and therefore also involved self-selection. Individuals were excluded if they were not currently practising or were unable to participate in an English-language interview.

### Recruitment

Interested individuals contacted the research team directly and were provided with a participant information statement and a consent form before an interview was arranged. Recruitment ceased after 13 interviews because subsequent interviews largely reinforced the principal patterns already identified regarding education, reactive recognition, interdisciplinary communication, and resource preferences. The research team judged that the combined dataset provided sufficient information power to address the exploratory study aim, while recognising that the small profession-specific samples did not support claims of saturation or generalisability [21, 22].

### Consent and enrolment

Potential participants were provided with a participant information statement and consent form before enrolment. Written informed consent was obtained electronically before the interview. The interviewer reconfirmed consent to participate and to audio-record the discussion at the beginning of each interview.

### Setting

Semi-structured interviews were conducted online using Microsoft Teams (URL: https://teams.live.com/v2/) between April and May 2026.

### Interview guide

An interview guide was developed by the research team (JP, AP, EK, AR) based on the study aims, relevant literature on medication-related oral health risks, pharmacist education, aged-care practice, and interdisciplinary care [11–17]. The draft interview guide was reviewed by researchers with expertise in pharmacy, dentistry, oral health, and qualitative research to assess content relevance, clarity, and alignment with the study objectives. Minor refinements to wording and question sequencing were made prior to data collection to improve clarity and flow. Questions were open-ended and supported by prompts exploring recognition of oral health issues, management strategies, interprofessional collaboration, referral pathways, and educational resource needs (Supplementary file 1).

### Data collection

Interviews were audio-recorded, transcribed, and de-identified using participant codes (e.g., Dentist 1–6 and Pharmacist 1–7). No repeat interviews were conducted, and transcripts were not returned to participants for comment or correction. An initial total sample of approximately 9 to 17 participants was anticipated to provide a range of relevant professional perspectives [23, 24]. The final sample size was determined pragmatically by considering information power, diversity of participants' experiences, the specificity of the study aim, and the depth and relevance of the interview data, rather than by a claim of thematic saturation.

### Data analysis

All interviews were transcribed verbatim using Microsoft Teams and manually checked for accuracy. JP conducted the initial familiarisation and coding of all transcripts. Codes, candidate themes, and interpretive decisions were reviewed through regular discussions with EK and AR. These discussions were used to challenge assumptions, refine theme boundaries, and consider alternative interpretations rather than to calculate inter-coder agreement [25–27]. Analysis involved familiarisation with the data through repeated reading of transcripts, followed by the generation of initial codes across the dataset. Codes were subsequently collated into potential themes, which were iteratively reviewed and refined in relation to the dataset. Themes and subthemes were then defined and named to reflect underlying patterns across participant accounts. The progression from an interview extract to an initial code, a candidate subtheme, and a final theme is provided in supplementary file 2. Interpretations and theme boundaries were refined through discussion and reference to the complete dataset.

### Ethics approval

This study was conducted in accordance with the principles of the Declaration of Helsinki. Ethics approval was granted by the University of Sydney Human Research Ethics Committee on 25 March 2026 (Project identifier: 2025/HE001328).

## Results

### Participants

A total of 13 participants were interviewed, comprising six dentists [D1–D6] and seven pharmacists [P1–P7] (Table 1). Interviews lasted approximately 30–45 min. Participants represented a broad range of practice settings, including community pharmacy, hospital pharmacy, medication review services, residential aged care, private dental practice, and corporate dental practice. Participants practised across five Australian states and territories: New South Wales (n = 4), Victoria (n = 3), Western Australia (n = 2), Queensland (n = 2), and the Australian Capital Territory (n = 2).

**Table 1:** Participant demographic and professional characteristics

Participant	Professional role	Primary practice setting	Years of experience	Geographical practice context	Frequency of experience with older adults, polypharmacy or residential aged care
Dentist 1	Dentist	Private dental practice	3 years	New South Wales Regional	Infrequently
Dentist 2	Dentist	Private dental practice	4 years	Victoria Regional	Frequently
Dentist 3	Dentist	Corporate dental practice	More than 2 years	Western Australia Metropolitan	Frequently
Dentist 4	Dentist	Rural dental practice	2–3 years	Western Australia Remote	Frequently
Dentist 5	Dentist	Private dental practice	Less than 1 year	Australian Capital Territory Metropolitan	Frequently
Dentist 6	Dentist	Corporate dental practice	Less than 1 year	Australian Capital Territory Metropolitan	Infrequently
Pharmacist 1	Pharmacist	Community pharmacy	14 years	Victoria Metropolitan	Frequently
Pharmacist 2	Pharmacist	Residential aged care and home medication review services	2–3 years	New South Wales Metropolitan	Frequently
Pharmacist 3	Pharmacist	Community pharmacy	Less than 1 year	Queensland Regional	Frequently
Pharmacist 4	Pharmacist	Hospital pharmacy and community pharmacy	More than 8 years	Queensland Regional	Frequently
Pharmacist 5	Pharmacist	Academic and clinical aged care practice	More than 10 years	Victoria Metropolitan	Frequently
Pharmacist 6	Pharmacist	Residential aged care and medication review services	More than 25 years	New South Wales Regional	Frequently
Pharmacist 7	Pharmacist	Medication review and community pharmacy practice	More than 27 years	New South Wales Metropolitan	Frequently

### Reflexive thematic analysis

Three major themes were identified relating to pharmacists’ role in recognising and managing medication-related oral health risks among older adults with polypharmacy: (1) pharmacists were increasingly encountering medication-related oral health risks despite limited formal preparation; (2) medication-related oral health risks were recognised reactively within fragmented systems of care; and (3) participants viewed pharmacists as potential contributors to earlier oral health intervention, although implementation required practical and workflow-integrated support (Table 2).

**Table 2:** Themes and subthemes identified from reflexive thematic analysis

Theme	Subthemes
Theme 1: Pharmacists were increasingly encountering medication-related oral health risks despite limited formal preparation	Limited formal oral health education Experiential and self-directed learning Increasing awareness through clinical exposure Limited preparation for interdisciplinary care
Theme 2: Medication-related oral health risks were recognised reactively within fragmented systems of care	Reactive and symptom-driven identification Xerostomia was recognised more readily than salivary gland hypofunction and its oral consequences Oral and functional consequences Fragmented communication and referral systems Structural barriers to oral healthcare Scope boundaries and escalation concerns
Theme 3: Participants viewed pharmacists as potential contributors to earlier oral health intervention, although implementation required practical and workflow-integrated support	Pharmacists as early identifiers of oral risk Preference for concise and practical resources Workflow integration and digital prompts Need for accessible and culturally appropriate resources Importance of clear referral thresholds and scope guidance Priority educational topics

The two professional groups contributed different but complementary perspectives. Pharmacists primarily described where medication-related oral health risks could be identified within medication review, dispensing, and aged-care workflows, including consideration of anticholinergic burden and communication with prescribers. Dentists primarily described the oral manifestations and downstream consequences they encountered after problems had developed, including candidiasis, caries, mucosal discomfort, swallowing difficulties, and delayed dental presentation. Dentists also identified concerns regarding referral thresholds and the risk of non-dental professionals acting beyond their scope. Together, these perspectives linked opportunities for earlier recognition of medication-related risks to the oral consequences and referral decisions observed in dental practice.

## Theme 1: Pharmacists were increasingly encountering medication-related oral health risks despite limited formal preparation

Pharmacists and dentists both described limitations in their formal preparation, although the areas of limited preparation differed between the two professions. While xerostomia was widely recognised across both professions, participants perceived broader medication-related oral complications, including candidiasis, denture-related problems, oral pain, mucosal irritation, dysphagia, and nutritional decline, as receiving comparatively little attention during undergraduate or postgraduate training.

Several pharmacists described oral health education as either minimal, incidental, or insufficiently connected to clinical practice. One pharmacist explained that prior to recent workplace exposure, oral health had received little meaningful attention within pharmacy education:

“If you asked me a month ago, I’d say nothing.” (Pharmacist 1)

Pharmacist 3, a community pharmacist with less than one year’s experience, described their formal preparation as minimal.

“I'd say very minimal formal training. I have been taught in university about oral health and what are potentially risks to the patients, but I wouldn't say there's been any formal training that I've completed that I have gone on those topics.” (Pharmacist 3)

Dentists also described limitations in formal preparation for interdisciplinary communication and aged-care complexity. Although participants commonly recalled education on dry mouth and medication side effects, this was frequently described as narrow and overly focused on symptom recognition rather than on broader systems-based management.

“Mainly about the relation between polypharmacy and xerostomia less so about candidiasis and mucosal irritation.” (Dentist 3)

Pharmacists and dentists described entering practice without adequate preparation for communicating across disciplines or navigating referral systems involving pharmacists, general practitioners, aged-care nurses, and dental professionals.

“There was little guidance about communicating with physicians or aged-care nurses.” (Dentist 2)

“A lot of the practical challenges in aged care aren’t fully addressed in formal training.” (Dentist 1)

Participants from both professions described relying heavily on experiential and self-directed learning to develop practical understanding of medication-related oral complications. Knowledge was commonly acquired through workplace exposure, interdisciplinary discussions, conferences, journal reading, and repeated encounters with medically complex older adults.

“Most of it for me has been informal learning, reading journals, looking things up myself.” (Pharmacist 7)

“Further understanding through… interdisciplinary discussions with medical and pharmacy teams.” (Dentist 2)

Several pharmacists explained that repeated exposure to frail older adults altered their understanding of the relationship between medications, oral health, and general well-being. One pharmacist described becoming increasingly aware of how oral problems contributed to reduced nutritional intake and quality of life among older adults.

“She has no appetite… and when she did try to eat, she finds that she can’t chew because she had no teeth.” (Pharmacist 2)

Pharmacists reflected that oral health complications were frequently overlooked within routine medication counselling despite their substantial impact on wellbeing.

“We talk about warfarin or blood thinners causing… bruising, but we never say… your gums could bleed.” (Pharmacist 7)

Both pharmacists and dentists described recognising links between medications, oral deterioration, and declining function that had not been emphasised during training. Dentists similarly described learning to recognise medication-related oral complications through repeated clinical exposure rather than formal education alone. Several participants explained that practical experience improved their ability to identify patterns linking medications to xerostomia, denture stomatitis, burning mouth symptoms, recurrent caries, and oral discomfort.

“Better recognise patterns—for example, linking denture stomatitis or burning mouth symptoms to medications.” (Dentist 3)

Across participant accounts, medication-related oral health risks were framed as an area that had received insufficient educational attention despite its perceived relevance to ageing populations and polypharmacy-related care.

## Theme 2: Medication-related oral health risks were recognised reactively within fragmented systems of care

Pharmacists and dentists both described medication-related oral health problems as being identified reactively, although pharmacists discussed recognition through medication review or symptom reporting, whereas dentists described patients presenting after oral symptoms or disease had developed. Xerostomia was the most commonly recognised medication-related symptom across participant accounts. Participants used terms such as ‘dry mouth’ and ‘xerostomia’, but generally did not clearly distinguish the subjective symptom of oral dryness from salivary gland hypofunction or reduced salivary flow.

“Pretty much all medicines cause dry mouth to some degree.” (Dentist 1)

“I had to have more in-depth understanding of which medication caused dry mouth because I see so many older people.” (Pharmacist 2)

Despite recognising dry mouth as common, dentists described oral complications as sometimes being overlooked until they became clinically significant. Several dentists explained that oral health problems were often identified only after symptoms had progressed substantially.

“The problem is often noticed after symptoms have already developed.” (Dentist 2)

One pharmacist described how oral deterioration among older adults could contribute to progressive nutritional and functional decline:

“The most important thing is that… she has no appetite… and when she did try to eat, she finds that she can’t chew because she had no teeth.” (Pharmacist 2)

“Some people have not seen dentists for like five, ten years.” (Pharmacist 7)

Pain and persistent symptoms were commonly described as major triggers for referral or escalation. Pharmacists frequently discussed encouraging patients to seek dental care only after symptoms became difficult to manage within pharmacy settings. A pharmacist described pain as a clear threshold for escalation but did not identify a comparable pathway for earlier or less severe symptoms.

“When there’s pain, you just have to see a dentist.” (Pharmacist 7)

Dentists similarly described referral escalation occurring when problems exceeded their perceived scope or became resistant to initial management strategies.

“When general management is not working or the case is complex and out of my scope.” (Dentist 3)

Both pharmacists and dentists described oral healthcare as poorly integrated within broader healthcare systems. Pharmacists and dentists described communication with general practitioners, nurses, and aged-care staff as fragmented, inconsistent, or indirect.

“Interdisciplinary care is also difficult as linking up with other health professionals is quite difficult.” (Dentist 1)

“As a pharmacist, we don’t have a lot of direct interaction with dentists.” (Pharmacist 2)

A pharmacist involved in medication review described uncertainty about whether oral health concerns should be directed to the resident’s general practitioner, dentist, nurse, or aged-care staff member.

“Who do you address these things to?” (Pharmacist 7)

Pharmacists also described structural barriers affecting oral healthcare access among older adults, including financial constraints, workforce shortages, geographic isolation, and limited availability of dental services in regional and remote areas.

“It’s hard when people don’t want to go or people can’t afford it.” (Pharmacist 1)

Pharmacists also emphasised the importance of maintaining professional scope boundaries while still supporting earlier recognition of oral complications.

“I don’t want to overstep… but I also don’t want to leave anyone undertreated.” (Pharmacist 1)

Dentists similarly expressed concern regarding simplified educational materials being used inappropriately without adequate clinical judgement.

“There’s a risk that staff may make clinical decisions outside their scope.” (Dentist 1)

## Theme 3: Participants viewed pharmacists as potential contributors to earlier oral health intervention, although implementation required practical and workflow-integrated support

One pharmacist emphasised that any resource would need to accommodate substantial time pressure in practice, commenting that ‘people are busy’. Pharmacists and dentists both preferred concise, quick-reference formats including checklists, flowcharts, decision trees, prompts, and one-page summaries.

“A one-page checklist would be the best and easiest to use.” (Dentist 3)

Pharmacists and dentists advocated for even shorter formats designed to minimise cognitive burden during dispensing or medication review activities.

“I wouldn’t even do one page… just go an A5.” (Pharmacist 1)

“A quick-reference table… allows for rapid consultation during clinical decision-making.” (Dentist 1)

“Helpful… to work through whether a problem can be managed locally or needs escalation.” (Dentist 2)

Participants also emphasised the importance of integrating oral health prompts into existing medication management systems and workflows. Pharmacists discussed the potential roles of electronic alerts, prescribing prompts, and embedded decision-support systems in identifying the risk of dry mouth and anticholinergic burden.

“As soon as two of them potentially cause it, then you’d get a giant prompt.” (Pharmacist 1)

Participants highlighted the importance of plain-language, visual, and culturally appropriate resources, particularly for residents with lower health literacy or cognitive impairment.

“Picture friendly… one page information sheet.” (Pharmacist 3)

“Use simple, plain language, have visual aids and ideally should have translated versions.” (Dentist 3)

Participants also emphasised the importance of clearly defining referral triggers, escalation pathways, and professional scope boundaries within educational materials. Many participants expressed concern that overly simplified resources could unintentionally encourage inappropriate self-management or practice outside professional scope.

“This is the absolute line… where you should go see [a professional].” (Pharmacist 1)

## Discussion

This study explored pharmacists' and dentists' perspectives on pharmacists' role in recognising and managing medication-related oral health risks in aged-care practice. Participants described limited formal preparation for medication-related oral health issues, fragmented referral pathways, and challenges in integrating oral health into routine medication management workflows. Pharmacists were viewed as well-positioned to contribute to earlier recognition of medication-related oral complications. Within a medication review, this role could involve asking about oral dryness, oral pain, swallowing difficulty, altered eating, recurrent oral infection, or recent dental deterioration; reviewing medicines associated with reduced salivary function; considering cumulative anticholinergic burden; documenting the concern; and communicating recommendations or referral needs to the general practitioner, dentist, nurse, resident, or carer. However, participants emphasised the need for practical educational resources, clearer interdisciplinary communication pathways, and workflow-integrated implementation strategies to support this role.

An important finding was that participants more readily discussed "dry mouth" or xerostomia than salivary gland hypofunction, despite these representing distinct concepts. Xerostomia refers to the subjective sensation of oral dryness, whereas salivary gland hypofunction is an objectively reduced salivary flow or function [28, 29]. Salivary gland hypofunction may occur without a reported sensation of dryness and is associated with an increased risk of dental caries, candidiasis, mucosal trauma, impaired chewing and swallowing, and nutritional decline [29, 30]. Educational resources for pharmacists should therefore distinguish patient-reported symptoms from objective or clinically suspected salivary dysfunction [29, 31]. Medicines with anticholinergic effects are particularly relevant, as cumulative anticholinergic burden may contribute to salivary gland hypofunction, particularly in older adults experiencing polypharmacy [4, 32]. Pharmacists can play an important role by identifying medicines that may impair salivary function, assessing cumulative anticholinergic burden during medication review, and communicating concerns to the general practitioner or other prescriber [33]. Where clinically appropriate, recommendations may include reviewing the ongoing indication for treatment, reducing the dose, deprescribing, or considering alternative medicines with lower anticholinergic activity [34, 35]. However, any medication changes should be individualised, taking into account multimorbidity, treatment stability, therapeutic priorities, and the potential risks of modifying otherwise effective therapy.

Participants’ accounts are consistent with previous literature suggesting that oral health is often poorly integrated into general healthcare and aged-care systems. Previous studies have similarly identified limited collaboration between pharmacists and dental professionals, as well as uncertainty regarding referral pathways and the scope of practice [36–38]. Research examining pharmacist-led aged-care initiatives, including the Aged Care On-site Pharmacist programme, has highlighted the complexity of implementation within residential aged-care settings and the need for stronger interdisciplinary structures to support medication safety and person-centred care [8–10]. Existing evidence also suggests that pharmacists frequently encounter barriers relating to communication, organisational variability, and workforce limitations, which may contribute to the delayed identification of medication-related oral complications [39]. The present study extends these findings by exploring how participants perceived broader systemic barriers as influencing the integration of oral healthcare into medication management practice.

These findings should also be interpreted in the broader context of oral health among older adults. Multimorbidity and polypharmacy may increase medication-related oral risk while simultaneously making medication changes more complex [4]. Financial disadvantage, limited dental insurance or public dental access, geographic isolation, transport barriers, cognitive impairment, and dependence on carers may also delay dental assessment [40, 41]. These factors may influence whether a pharmacist’s identification of an oral health concern results in timely assessment or treatment.

Participants’ preference for concise, workflow-integrated educational resources also aligns with implementation science and adult-learning literature. Participants strongly favoured practical tools such as one-page checklists, quick-reference tables, electronic prompts, and decision-support aids that could be rapidly accessed during clinical practice [42, 43]. Previous studies have shown that healthcare professionals are more likely to engage with case-based, clinically relevant, and adaptable educational interventions [44, 45]. Similarly, research exploring oral health education for pharmacists has identified the importance of practical learning formats, interdisciplinary education, and culturally appropriate communication strategies [11, 46].

### Implications for practice and education

Educational initiatives should prioritise concise, clinically relevant resources that integrate into medication reviews and Aged Care On-site Pharmacist services. Strengthening pharmacist education in salivary gland hypofunction, xerostomia, medication-related oral disease, anticholinergic burden, referral thresholds, and communication with prescribers and dental professionals may support pharmacists’ involvement in recognising and communicating medication-related oral health risks [11]. Embedding oral health considerations into routine medication management may help pharmacists communicate medication-related oral health concerns with dental professionals and other care providers [13, 36].

### Strengths and limitations

Several limitations should be considered when interpreting these findings. The study involved a relatively small purposive sample of pharmacists and dentists practising in Australia, which may limit the transferability of the findings to other healthcare settings or professional groups. The study did not include general practitioners, nurses, speech pathologists, aged-care workers, residents, or family carers, all of whom may provide important perspectives on the recognition, assessment, and referral of medication-related oral health problems within aged-care settings [47, 48]. Although nurses and aged-care staff were frequently discussed by participants, they were not interviewed directly. Recruitment through professional networks and social media may also have introduced elements of convenience and self-selection despite the purposive eligibility criteria. In addition, some dental participants had limited direct exposure to residential aged care or older adults experiencing polypharmacy. Their perspectives may therefore reflect broader experiences in general dental practice rather than specialised aged-care practice.

## Conclusion

This study explored pharmacists’ and dentists’ perspectives on pharmacists’ role in recognising and managing medication-related oral health risks in aged-care practice. The inclusion of both professions showed that pharmacists and dentists viewed different stages of the same clinical pathway. Pharmacists described opportunities to identify medicine-related risk earlier, whereas dentists described the oral consequences and delayed presentations that followed when risks were not recognised or addressed. Participants described limited formal preparation, fragmented interdisciplinary communication, unclear referral pathways, and poor integration of oral health within medication management workflows. Participants preferred concise, practical, and workflow-integrated resources to support pharmacists in recognising, referring, and communicating medication-related oral health risks. Embedding such resources within medication review and Aged Care On-site Pharmacist workflows may support pharmacists’ involvement in recognising and communicating oral health concerns among older adults with polypharmacy.

## Supplementary Information

Below is the link to the electronic supplementary material.

Supplementary file1 (DOCX 22 KB)

Supplementary file2 (XLSX 62 KB)

## Data Availability

The datasets generated during and/or analysed during the current study are available from the corresponding author on reasonable request. All data supporting the findings of this study are available within the paper and its Supplementary files.
