# Supplementary material for: Pharmacists’ role in recognising medication-related oral health risks in aged care: a qualitative study of pharmacists’ and dentists’ perspectives
Source: Int J Clin Pharm. 2026 Sep 16;48(6):2479–89. doi: 10.1007/s11096-026-02225-w (PMC13731708; doi:10.1007/s11096-026-02225-w)
Supplement: Supplementary file 1 [file 11096_2026_2225_MOESM1_ESM.docx]

**Submission ID -** 66b14314-e573-4779-a864-e397dad84b51

**Article title -** Pharmacists' role in recognising medication-related oral health risks in aged care: A qualitative study of pharmacists' and dentists' perspectives
**Journal -** International Journal of Clinical Pharmacy
**Authors -** Joon Soo Park, Amy T Page, Estie Kruger, Woosung Sohn, Bradley Christian, Arash Rudman
**Corresponding author:** Joon Soo Park [School of Health and Clinical Sciences, The University of Western Australia, Crawley, WA, Australia]
**Email:** alex.park@uwa.edu.au

**Supplement file 1 - Semi-structured interview guide**

Participant context

1. Please describe your current role and primary practice setting.
2. Approximately how many years have you worked in this area?
3. How often does your work involve older adults with polypharmacy or residential aged-care settings?

Current and prior education

1. What have you been taught, formally or informally, about medication-related oral health risks (e.g., dry mouth, candidiasis, mucosal irritation)?
2. Which aspects of this education were useful in practice, and which were not?
3. What have you learned through clinical experience that was not covered during training?

*Probes*

- Timing and setting of training
- Relevance to aged-care practice
- Examples of learning transferred into practice
- Educational gaps identified early in practice
- Assessments, credentials, or continuing professional development activities completed

Encounters in practice

1. Can you describe a recent situation where medication contributed to an oral health problem for an older person? How was it managed?
2. Where do these risks most commonly arise within your workflow (e.g., prescribing, dispensing, HMR/RMMR/ACOP, case conferences, dental visits)?
3. When is referral to a dentist or other clinician triggered, and what factors make referral easier or more difficult?

*Probes*

- Dry mouth screening
- Anticholinergic burden
- Denture-related issues
- Oral candidiasis
- Analgesic, benzodiazepine, or opioid-related concerns
- Communication with carers and families

Equity, safety, and person-centred care

1. How should educational materials support person-centred care for older adults, including people with dementia, culturally and linguistically diverse (CALD) communities, and those in rural or remote settings?
2. What safety concerns should be anticipated (e.g., misuse, scope boundaries, documentation issues)?

*Probes*

- Use of plain-language materials for patients and carers
- Role of interpreters for CALD communities
- Alignment with deprescribing initiatives
- Escalation pathways and referral responsibilities
- Strategies to minimise fragmented care across providers

Preferred formats for educational materials

1. If you had to choose one or two formats you would realistically use in a busy clinical setting, what would they be and why?
2. What length and level of detail would be most practical?

*Probes*

- One-page checklists
- Decision trees
- Case vignettes
- Micro-learning resources
- Quick-reference tables
- Printable versus mobile formats
- Integration within HMR/ACOP templates
- Visual aids and mnemonics
- Use before, during, or after reviews

Priority topics and essential content

1. Which topics should be prioritised to improve aged-care practice?
2. For each topic, what information should be included?

*Prompts*

- Dry mouth: recognition, causative medicines, prevention and management, referral triggers
- Anticholinergic burden: thresholds, screening tools, deprescribing prompts, safer alternatives
- High-risk medication classes and oral adverse effects
- Referral criteria and interprofessional communication expectations
- Patient and carer education strategies
- Monitoring, follow-up, and documentation requirements

Usability and workflow integration

1. What would make these materials easy to integrate into practice?
2. What barriers might limit their use?

*Probes*

- Preferred platforms (e.g., LMS, PDF, intranet, clinical software)
- Searchability and accessibility
- Integration with existing forms and templates
- Resource ownership and endorsement
- Frequency of updates
- Accessibility for rural and remote practitioners

Existing resources and current gaps

1. What resources do you currently use in this area, and where do they fall short?
2. If you could improve one aspect of current guidance, what would it be?

*Probes*

- Therapeutic Guidelines
- Local protocols
- Professional society resources
- Ad hoc checklists
- Navigation and usability challenges
- Inconsistencies between professions

Implementation requirements

1. What would support adoption of these educational materials within your workplace or service?
2. Which people, organisations, or professional bodies would need to support implementation?

*Probes*

- Professional organisations (e.g., PSA, ADA)
- Aged-care providers
- Education programme leads
- Software vendors
- Continuing professional development alignment
- Micro-credentialling opportunities

Role-specific questions

*Pharmacists*

1. Where within HMR/RMMR/ACOP workflows would dry mouth or anticholinergic prompts be most useful?
2. What information or thresholds would you want included on a quick-reference resource?
3. What referral information from dentistry would support continuity of care?

*Dentists*

1. What information from pharmacists would improve dental management?
2. Which medication-related oral conditions should pharmacists prioritise screening for?
3. What information should be included in referrals from pharmacists?

Closing question

“Is there anything we have not discussed that you believe should be included?”
